# Red: an intelligent, rapid, accurate tool for detecting repeats de-novo on the genomic scale

**DOI:** 10.1186/s12859-015-0654-5

**Published:** 2015-07-24

**Authors:** Hani Z. Girgis

**Affiliations:** 1 Computational Biology Branch, National Center for Biotechnology Information, National Library of Medicine, National Institutes of Health, 8600 Rockville Pike, Bethesda, 20894 MD USA; 2 Tandy School of Computer Science, University of Tulsa, 800 South Tucker Drive, Tulsa, 74104 OK USA

## Abstract

**Background:**

With rapid advancements in technology, the sequences of thousands of species’ genomes are becoming available. Within the sequences are repeats that comprise significant portions of genomes. Successful annotations thus require accurate discovery of repeats. As species-specific elements, repeats in newly sequenced genomes are likely to be unknown. Therefore, annotating newly sequenced genomes requires tools to discover repeats de-novo. However, the currently available de-novo tools have limitations concerning the size of the input sequence, ease of use, sensitivities to major types of repeats, consistency of performance, speed, and false positive rate.

**Results:**

To address these limitations, I designed and developed Red, applying Machine Learning. *Red is the first repeat-detection tool capable of labeling its training data and training itself automatically on an entire genome.* Red is easy to install and use. It is sensitive to both transposons and simple repeats; in contrast, available tools such as RepeatScout and ReCon are sensitive to transposons, and WindowMasker to simple repeats. Red performed consistently well on seven genomes; the other tools performed well only on some genomes. Red is much faster than RepeatScout and ReCon and has a much lower false positive rate than WindowMasker. On human genes with five or more copies, Red was more specific than RepeatScout by a wide margin. When tested on genomes of unusual nucleotide compositions, Red located repeats with high sensitivities and maintained moderate false positive rates. Red outperformed the related tools on a bacterial genome. Red identified 46,405 novel repetitive segments in the human genome. Finally, Red is capable of processing assembled and unassembled genomes.

**Conclusions:**

Red’s innovative methodology and its excellent performance on seven different genomes represent a valuable advancement in the field of repeats discovery.

**Electronic supplementary material:**

The online version of this article (doi:10.1186/s12859-015-0654-5) contains supplementary material, which is available to authorized users.

## Background

We live in exciting times. Soon, we will witness the sequencing of genomes of thousands of species. Significantly, our knowledge of repetitive DNA, or repeats, an important component of the genomes of almost all species, will expand. Repeats may make up a large percentage of a genome. For example, it has been estimated that the percentage of repeats in the human and the maize genomes are 50 % [1] and 85 % [2]. Because repeats are species specific, repeats of the majority of newly sequenced genomes are unknown. Therefore, methods that can efficiently locate repeats de-novo without relying on known repeats play a crucial role in annotating newly sequenced genomes.

Repeats are very important clinically. Up until 2012, insertions of non-LTR retrotransposons were known to be responsible for 96 human diseases including colon cancer, breast cancer, leukemia, cystic fibrosis, hemophilia, muscular dystrophy, and chronic granulomatous disease [3–5]. Microsatellites (MS), one type of tandem repeats, are also linked to several diseases such as fragile X syndrome, Huntington’s disease, Kennedy’s disease, myotonic dystrophy, and triplet-repeat expansion diseases [6]. Further, MS have several biomedical applications in such processes as DNA paternity testing and forensic DNA finger printing [7].

Scientists have recognized the multiple molecular functions of repeats since the fifties. Saha et al. highlight some known functions of transposable elements (TE) including their role in gene expression [8]. Interestingly, McClintock observed that, when a genome is under stressed conditions, transposons can modify actions of regular genes and “restructure the genome at various levels” [9]. Furthermore, “multiple transpositions” of transposons provide a mechanism to generate new genes [10]. Tandem repeats (TR), especially MS, have several functions involving gene regulation and recombination [6].

It has been reported in the Bioinformatics field that masking TR improves the quality of alignments produced by alignment algorithms [11]. These algorithms represent essential tools for studying Molecular Biology. Additionally, the time required to search for a non-repetitive sequence, e.g. a coding region, in a genome can be reduced dramatically by excluding repeats from the search database.

TR and TE comprise two main classes of repeats. Regarding the first class, TR consist of a repeated short motif(s) in tandem. Microsatellites, minisatellites, satellites, and low complexity regions are the main classes of TR. As for the second class, TE were first discovered in the maize genome by McClintock, who described them as “mutable genes” or “unstable genes” [12]. Autonomous TE carry genes needed for their transposition. TE are found in a large number of copies interspersed throughout the genome. TE include DNA transposons and retrotransposons. DNA transposons relocate via a “cut and paste” mechanism, whereas retrotransposons relocate via a “copy and paste” mechanism involving RNA as the intermediate molecule. Lengths of TE range from tens to thousands of nucleotides. Sequences of TE include several features that are characteristic of each class of transposons.

Many computational tools have been developed to detect TE since 1994. Several of these tools are reviewed in [13–17]. Here, I present a slightly modified version of the classification by **Lerat** [16]. Computational methods for locating TE are classified into the following six categories:

- Library-based methods: RepeatMasker (http://www.repeatmasker.org) and Censor [18] are widely used library-based tools. A library-based tool searches a sequence for copies of known repeats collected in a database such as RepBase [19]. RepBase comprises a library of manually annotated repeats in eukaryota.
- Learning-based methods: A method developed by Andrieu et al. [20] assumes that the nucleotide composition of TE is different from that of the rest of the genome. This assumption is supported by the fact that the genes of TE are different from regular genes. Developing methods within this category involves applying Machine Learning algorithms to obtain models that distinguish sequences of TE from non-TE sequences.
- Signature-based methods: Each class of TE has a set of unique features such as a target site duplication, a poly-A tail, terminal inverted repeats, long terminal repeats, and/or a hairpin loop. The signature of a class of TE consists of a subset of these features. A signature-based tool searches a sequence for features comprising the signature of the class of interest [21–24].
- Comparative-genomics-based methods: TE are species specific. Therefore, when two genomes of closely related species are compared, TE are expected to be found in one genome and absent in the other. Long gaps in the pair-wise alignment mark potential TE [25]. Similarly, still-transposing TE can be detected by comparing the genomes of different individuals of the same species [3].
- De-novo methods: The repetitive nature of TE inspired the emergence of de-novo methods. De-novo methods depend on one of two processes: “self-comparison” or counts of k-mers. Self-comparison methods align a genome, or samples of it, versus itself (e.g. ReCon [26] and PILER [27]). Examples of tools that count exact or approximate (known as “spaced”) k-mers are ReAS [28], RepeatScout [29], WindowMasker [30], Repseek [31], and Tallymer [32].
- Consensus methods: These methods combine TE located by a group of different tools. For example, a pipeline consisting of library-based methods, learning-based methods, and de-novo methods has been proposed [33]. The REPET pipeline [34] utilizes de-novo and/or signature-based methods. RepeatModeler (http://www.repeatmasker.org) is based on ReCon and RepeatScout for the identification of TE; additionally, it applies TRF [35], which is a de-novo tool used for locating TR.

Detecting TR has received equal attention. Numerous computational tools have been developed for this purpose, in particular for detecting microsatellites, an important class of TR. Several of these tools are reviewed in [36–39]. Tools for detecting TR fall into three categories similar to those of TE. The first category involves library-based tools (e.g. RepeatMasker). The second includes learning-based tools (e.g. MsDetector [40]). The third, and final, category comprises de-novo tools (e.g. TRF [35], mreps [6], STAR [41], and TANTAN [11]). A widely used tool for masking low-complexity regions de-novo is DUST [42].

Although repeats are abundant in the majority of genomes, “the algorithms and computational tools for identifying and studying repeat sequences are relatively primitive compared to those being utilized to explore genes,” as Saha et al. pointed out [14]. In another study, Saha et al. [15] identified the following limitations of the currently available tools: (i) the majority of these tools cannot process a complete chromosome, let alone a whole genome; (ii) the processing time may be long and unpractical; and (iii) there are difficulties in installing and using some tools.

The goal of my study is to design and develop a tool that addresses the above mentioned limitations. Specifically, the new tool should be capable of processing a whole genome in a reasonable amount of time without relying on other tools. In addition, due to the important role of de-novo methods in annotating new genomes, the new tool must rely only on the repetitive nature of repeats. To this end, I have designed and developed a computational tool I call Red (REpeat Detector) that accomplishes the stated goals.

The input to Red comprises the sequences of the genome of interest. Red can process assembled as well as unassembled genomes. To start, Red searches for k-mers that are repeated at least three times in the genome and their counts are greater than what could be expected by chance in a genome of similar composition. Then an analytical method is applied to locate candidate repetitive regions consisting mainly of the repeated k-mers. These candidate repetitive regions and the potential non-repetitive regions are used for training a hidden Markov model (HMM) that scans the whole genome searching for repeats. The process of training the HMM is an instance of supervised learning that traditionally requires manually annotated data. Because Red has the ability to label candidate repetitive regions and potential non-repetitive regions, it does not require manually annotated data. Moreover, the labeling and the training processes are carried out automatically on each genome. To the best of my knowledge, *Red is the first repeat-detection tool that has the capability of labeling its own training data and can train itself automatically on each genome.*

My assessment of Red and three related tools on seven genomes demonstrate that Red has the following nine advantages:

- Red is easy to install and use because it does not depend on other tools;
- Red is sensitive to both TE and TR, while the related tools are either primarily sensitive to TR or TE, but not both;
- Red performs well on the seven tested genomes, while the other tools perform well on some of the genomes but not all of them;
- Red is much faster than some of the widely used de-novo tools;
- Red has a low false positive rate;
- Red is capable of processing a genome that has an unusual nucleotide composition while achieving a high sensitivity and maintaining a moderate false positive rate;
- Red can discover a large number of novel repetitive segments;
- Red has the ability to discover repeats in bacterial genomes; and
- Red is capable of detecting repeats in unassembled genomes.

## Methods

I designed and developed Red (REpeat Detector), a de-novo tool for discovering repetitive elements in DNA sequences comprising a genome. Red utilizes a hidden Markov model (HMM) dependent on labeled training data, i.e. it is an instance of supervised learning. Traditionally, the training data are based on manually annotated sequences. However, this is not the case with Red. Red identifies candidate repetitive regions using (i) adjusted counts of k-mers, (ii) a signal processing technique, and (iii) the second derivative test. These candidate regions are used for training the HMM. To the best of my knowledge, *Red is the first repeat-discovery system that has the ability to generate its own labeled training data and to train itself automatically on an input genome.*

The input to the system is sequences, in FASTA format, comprising an assembled or an unassembled genome. Red outputs the genomic locations of the candidate regions and the final repeats found in the genome as well as the masked sequences. Red consists of the following four modules: (i) the scoring module, (ii) the labeling module, (iii) the training module, and (iv) the scanning module.

### The scoring module

The input to this module is a set of sequences of nucleotides A, C, G, and T. The module outputs the corresponding sequences of scores. The score of a nucleotide is the adjusted count of a word of length *k* (also known as a k-mer) starting at this nucleotide. Next, I discuss an efficient hash table used for counting k-mers and adjusting their counts.

#### Efficient hash table

I utilized a hash table, that is a data structure, and a time-efficient hash algorithm to store the adjusted counts of all DNA words of length k. In theory, a hash table has two columns. Each row in the table represents a key-value pair. In practice, a hash table is implemented as an array, i.e. a single-column table. Values are stored in the cells of the array. To access the value associated with a key, a hash function calculates a unique number that is the index of the value in the array. There are many hash functions available; however, these functions are not designed to handle long sequences of DNA. Standard hash functions calculate the index of one key at a time. In contrast, the hash function, which I designed for Red, efficiently calculates the indices of thousands, even millions, of consecutive words, i.e. keys, in a DNA sequence. Specifically, the index of a k-mer is the quaternary number obtained by converting the nucleotides A, C, G, and T to the digits 0, 1, 2, and 3, respectively. The quaternary numbers can be computed efficiently for a sequence of adjacent words in a chromosome using Horner’s rule [43]. This data structure takes advantage of the large memory capacity that is available nowadays in personal computers. As for run time, updating the table and retrieving values from it are very time-efficientoperations.

#### Completing the table

The scoring module scans the set of sequences comprising the genome, sequence by sequence. As the module scans a sequence nucleotide by nucleotide, it updates the count of the word starting at a nucleotide in the table. Once the scanning of the genome is complete, entries in the table represent the observed counts for all the words in the genome. Then, the observed counts are adjusted. To avoid coding regions and duplicated segments, the adjusted count of a k-mer that occurs once or twice is zero. Similarly, the adjusted count is zero if the observed count is less than or equal to the expected count that is calculated using a Markov chain trained on the same genome. Finally, if the observed count of a k-mer is greater than the expected count and it occurred at least three times, the adjusted count is the count of the k-mer observed in the input genome minus the expected count. The rational for adjusting the counts was inspired by the idea of “correct[ing] for biases in sequence composition” [31]. At this point, the table is ready to be used for scoring input sequences.

#### Scoring a sequence

**Fig. 1:**
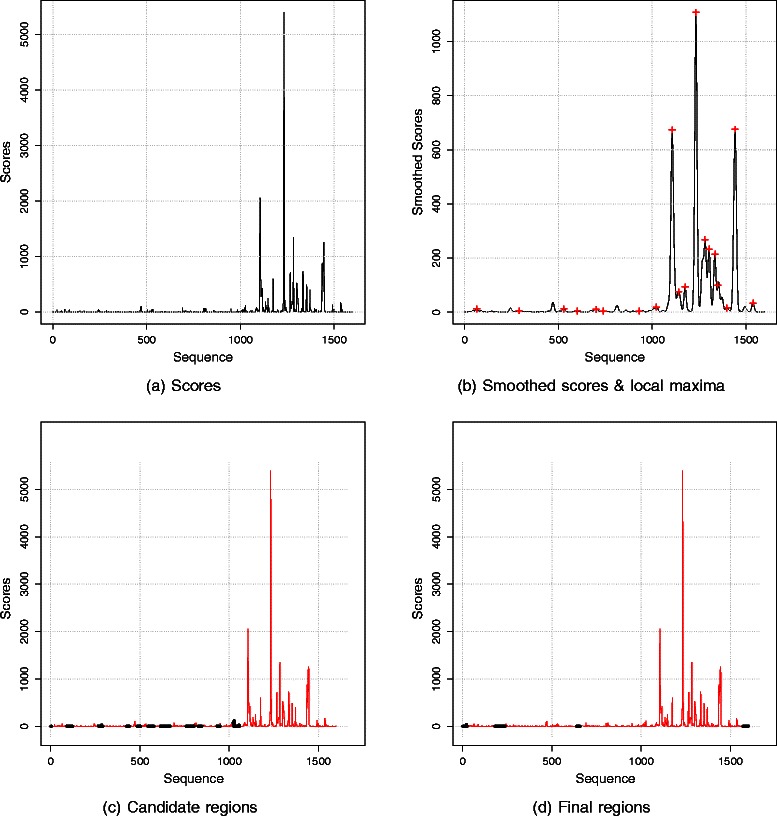
Method overview. **a** A sequence of scores: The score of each nucleotide is the adjusted count of the k-mer starting at this nucleotide. **b** Smoothed scores: The smoothed score is the weighted average of the flanking scores. The weights are assigned according to a Gaussian distribution. The local maxima, marked by ‘+’, are located using the second derivative test. **c** Candidate regions: The labeling module locates candidates (thin and colored in red) and potential non-repetitive regions (thick and colored in black). Regions found in the whole genome are used for training the hidden Markov model (HMM). **d** Final regions: The scanning module applies the trained HMM to locate the final repetitive regions (thin and colored in red). Notice that the final repetitive regions are less fragmented than the candidates. Additionally, they include all local maxima even the ones that were missed by the labeling module

The scoring module scans the input sequence, nucleotide by nucleotide. For each word starting at a nucleotide, the module finds the adjusted count of the word in the table. Subsequently, the adjusted count of this word is appended to the end of the output sequence as the score representing this nucleotide. Figure 1(a) provides an example sequence of scores calculated by the scoring module.

Next, I explain how scores are used for labeling candidate repetitive regions and non-repetitive regions. In this article, I refer to potential repetitive regions as “candidates.” The training module uses the candidates and the potential non-repetitive regions for training the HMM.

### The labeling module

The labeling module searches the score sequences for candidate repetitive regions. Once the search is complete, candidate repetitive regions and non-repetitive regions are listed. To this end, the following three steps are executed in order.

#### Step 1 - Smooth the score sequence using a Gaussian mask

Repetitive regions are likely to consist mainly of high scores; in contrast, non-repetitive regions are likely to consist mainly of low scores. In the current study, it has been observed that high scores in the score sequence appear close to each other and low scores occur close to each other. However, low scores may be found in regions consisting mainly of high scores and vice versa. Therefore, it is more informative to represent a score as the weighted average of the flanking scores. The weights are assigned according to a Gaussian distribution (Equation 1), in which the closer the neighboring score to the score of interest is, the higher its weight is.

(1) $$  g(x) = \frac{1}{\sqrt{2\pi\sigma^{2}}} \exp\left\lbrace -\frac{(x - \mu)^{2}}{2\sigma^{2}}\right\rbrace  $$

**Fig. 2:**
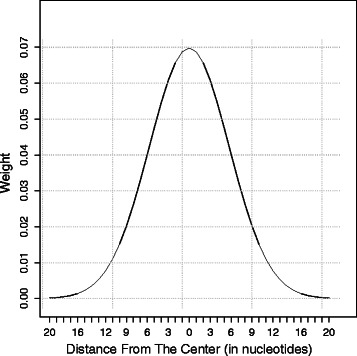
Gaussian mask. This example mask represents the weights used by the labeling module for smoothing a sequence of scores. The width of this example mask is 40. Therefore, the smoothed score is the weighted average of the scores of the 40-bp-long region centered on the score of interest

In this equation, *μ* and *σ* are the mean and the standard deviation of the Gaussian distribution. Precomputed weights calculated according to a Gaussian distribution with a specific mean and a specific standard deviation are called a mask. The width of a mask is 7×*σ* because almost all samples fall within 3.5 standard deviations on each side of the mean. Figure 2 demonstrates an example mask. To smooth a score, the mask is centered on this score; then the weighted mean is calculated according to Equation 2.

(2) $$  \overline{s_{i}} = \sum_{j = i-h,p=1}^{i+h, 2h+1} s_{j} m_{p} \div \sum_{p=1}^{2h+1} m_{p}  $$

The symbol *m* is the sequence of the weights of the mask, $\overline {s_{i}}$ is the smoothed score, and *s*_*i*_ is the original score. Figure 1(b) displays the result of smoothing the scores shown in Fig. 1(a).

#### Step 2 - Determine local maxima analytically

Local maxima are likely to occur within repetitive regions because mainly high scores comprise these regions. The second derivative test is used for determining local maxima in a sequence of smoothed scores. Approximations of the first and the second derivatives are used in the calculation of this test. These approximations are calculated by implementing Equations 3 and 4 [44].

(3) $$\begin{array}{*{20}l}  f'(s_{i}) &= \sum_{j = i-w}^{i-1} s_{j} - \sum_{j=i+1}^{i+w} s_{j} \end{array} $$

(4) $$\begin{array}{*{20}l}  f^{\prime\prime}(s_{i}) &= \sum_{j = i-w}^{i-1} s_{j} + \sum_{j=i+1}^{i+w} s_{j} - 2 w s_{i} \end{array} $$

Here, *w* is a small window (10 is currently used). The approximation of the first derivative is the difference between the summation of the smoothed scores of the window, which consists of *w* nucleotides, preceding the nucleotide of interest and that of the window following the nucleotide. The approximation of the second derivative is the difference between the summation of the smoothed scores of the preceding and the following windows and 2*w* times the smoothed score of the nucleotide of interest. The second derivative test states that a local maximum occurs at a point if two conditions are met: (i) the value of its first derivative is zero, and (ii) the value of its second derivative is negative. In practice, the value of the first derivative is considered zero if the sign (+ / −) of the first derivative changes between two consecutive points because the zero is crossed between these two points. Figure 1(b) shows the local maxima marked by the ‘+’ sign, identified in the sequence of smoothed scores.

#### Step 3 - Delineate candidates and potential non-repetitive regions

A sequence consists of repetitive regions separated by non-repetitive regions. The presence of local maxima and of high scores is characteristic of repeats, whereas non-repetitive regions consist mainly of low scores that do not include a local maximum. The core of a candidate repetitive region, a sequence of high scores with at least one maximum, is expanded step-wise until a non-repetitive region is encountered. The details of executing this step are given in the Additional file 1. Figure 1(c) provides an example of the labeled regions.

Next, the training module trains the HMM on the labeled regions. Once the HMM is trained, the scanning module searches for repeats in the genome.

### The training module

Candidate regions located by the labeling module may have inaccurate boundaries. If the width of the mask is small, some regions may be fragmented (see Fig. 1(c) for an example). In contrast, if the width of the mask is large, some regions may represent two or more separate repetitive regions merged together including interleaving non-repetitive regions. Therefore, an additional correction step is needed. Such a correction step can be carried out using a probabilistic model. Although candidate regions may include noisy regions, a large percentage of these candidates are repeats or fragments of repeats. Accordingly, these regions can be used for training a probabilistic model that is tolerant of noise in training data. A probabilistic model such as an HMM trained on the labeled repetitive and non-repetitive regions should delineate repeats more accurately (compare Fig. 1(c) to Fig. 1(d)).

#### Scores as time-series data

In a time series, an observed event depends on the preceding events in the series. Repetitive regions consist mainly of high scores as well as a small percentage of low scores. In contrast, non-repetitive regions consist mainly of low scores in addition to a small percentage of high scores. These properties suggest that a score depends on the preceding scores. Therefore, a series of scores can be considered a time series. Consequently, one can guess whether a score of a nucleotide is high or low by examining the preceding scores. In the majority of cases, the guess is correct. In other cases, when the guessed value does not agree with the actual score, the incorrect guess is regarded as a bridge over a gap in the actual scores (if the region is repetitive) or as a noise filter (if the region is non-repetitive).

#### Hidden Markov models

HMMs [45] are well suited for time-series data. An HMM consists of a set of states and three types of probabilities. A state generates a set of observations. An HMM is based on prior probabilities, transition probabilities, and output probabilities. The prior probability of a state is the probability that it is the first one in a series. The transition probability between two states – *S*_*a*_ and *S*_*b*_ – is the probability that the next state is *S*_*b*_ when the previous state is *S*_*a*_. The probability that a state generates a specific observation is known as the output probability.

#### Model design

**Fig. 3:**
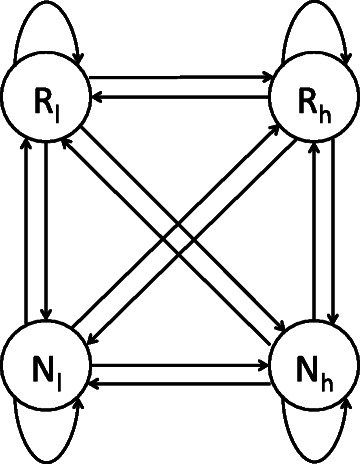
Example of the HMM structure. In this simplified example, the HMM consists of four states: two states representing repeats (R _*l*_ and R _*h*_) and two states representing non-repeats (N _*l*_ and N _*h*_). The model has transitions from each state to the other three states. Additionally, there is a transition from each state to itself to allow the model to stay in the same state that generates multiple subsequent scores. The assumption underlying this structure is that repetitive regions consist mainly of high scores interleaved with a small number of low ones; in contrast, non-repetitive regions consist mainly of low scores interleaved with a small number of high ones. States R _*h*_ and R _*l*_ generate high and low scores in repetitive regions. States N _*h*_ and N _*l*_ generate high and low scores in non-repetitive regions

Half of the HMM states are designed to generate scores, high and low, of repetitive regions, whereas the other half of the states are designed to generate scores, high and low, of non-repetitive regions. Figure 3 shows the structure of a simple HMM with four states. Two of the states generate low and high scores in repetitive regions, and the other two generate low and high scores in non-repetitive regions. The model has transitions from each state to the other three states. In addition, there is a transition from a state to itself, i.e. the model may stay in the same state to generate multiple subsequent scores. The actual HMM has a similar structure; however, it has a larger number of states. A state in the HMM is designed to generate a specific range of scores that have the same logarithmic value (Equation 5).

(5) $$ \text{output(s)} = \left\{ \begin{array}{ll} \lceil {log}_{t}(s) \rceil &\text{if s \(>\) 0} \\ 0 &\text{if s \(\le\) 0} \end{array} \right.   $$

The base of the logarithmic function, *t*, is the threshold of the low scores used for defining non-repetitive regions; see the Additional file 1. Each observed score can be generated by one of two states: a repetitive state or a non-repetitive state. Accordingly, the total number of states is twice the logarithmic value of the maximum score found in the genome of interest.

#### Learning probabilities

Training the HMM requires determining the prior, the transition, and the output probabilities. These probabilities can be calculated from the labeled candidate and non-repetitive regions. The prior probabilities are calculated by counting the first state of a candidate or of a non-repetitive region and dividing the count of each state by the total number of regions. The output probabilities are always 1 because a state is designed to generate scores that have the same logarithmic value. Calculating the transition probabilities involves building a matrix that has the same number of rows and columns. Each entry in the matrix represents the probability of moving from a state represented by the row name to a state represented by the column name. To calculate the transition probability from a state *a* (*S*_*a*_) to a state *b* (*S*_*b*_), this module counts the times *S*_*b*_ occurred after *S*_*a*_, and then divides the count by the total number of transitions out of *S*_*a*_. Again, *a* and *b* are the logarithmic values of the scores according to Equation 5. Each logarithmic value can represent one of two states according to its location in a candidate region or a potential non-repetitive region. Note that the matrix is not symmetric, i.e. the transition probability from *S*_*a*_ to *S*_*b*_ is not the same as the transition probability from *S*_*b*_ to *S*_*a*_.

### The scanning module

The scoring module generates the sequence of scores of the input nucleotide sequence. The series of states that are likely to generate the sequence of scores are determined by Viterbi’s algorithm using the trained HMM. The locations of adjacent scores that have repetitive states are then determined. The corresponding sequences of nucleotides represent the final repetitive elements. The end of a repetitive region is adjusted to include the adjacent *k*−1 nucleotides. Recall that *k* is the size of the k-mer, and the right *k*−1 nucleotides are part of the final k-mer of a repetitive region. Examples of regions located by the scanning module are shown in Fig. 1(d).

### Supplementary information

Up to this point, I illustrated the scoring module, the labeling module, the training module, and the scanning module. More information about the methodology of Red is available in the Additional file 1. In that section, I provide the details of how the labeling module delineates candidate repetitive regions and potential non-repetitive regions. Then I show that the run time of Red is linear with respect to the genome size. Additionally, I discuss the default values of Red’s parameters. Then, I give the details of the related tools. Finally, I list the sources of the data used in this study.

### Availability

The C++ source code and Red binaries for Unix 64-bit and Mac 64-bit are available as Additional files 2, 3 and 4.

## Results

In this section, first, I define the criteria to evaluate Red and the three related tools. Then I discuss the contributions of this study and the advantages of Red over the currently available tools.

### Evaluation measures

The following criteria were used in this study to evaluate Red, RepeatScout, ReCon, and WindowMasker: Sensitivity (SN), Specificity (SP), Percentage Predicted (PP), False Positive Length (FPL), Potential Repeats (PR), Time, and Memory. The majority of these measures, or their derivatives, provide standard evaluation criteria and have been used in previous studies [15, 16, 40].

The sensitivity (SN) of a tool is evaluated on the basis of the repeats located by RepeatMasker. RepeatMasker is considered the standard tool for detecting repeats because it depends on a manually annotated library of repeats called RepBase. Although repeats found by RepeatMasker are not perfect, they are the best available comprehensive set of repeats. Recall that Red, RepeatScout, ReCon, and WindowMasker are de-novo tools, whereas RepeatMasker is a knowledge-based tool. Accordingly, the sensitivity of a tool to the repeats detected by RepeatMasker is a predictor of the ability of that tool to discover repeats de-novo. Equation 6 defines the sensitivity.

(6) $$  {SN}_{class} = 100 \times \frac{O}{R}  $$

In this equation, *O* is the overlap between the repeats predicted by a tool and the repeats detected by RepeatMasker; *R* is the length of the repeats detected by RepeatMasker. The length is measured in base pair (bp). Repeats detected by RepeatMasker belong to a specific class. SN _*te*_ is the sensitivity to all types of transposons. SN _*tr*_ is the sensitivity to tandem repeats including microsatellites and satellites. SN _*low*_ is the sensitivity to low complexity regions. SN _*other*_ is the sensitivity to other kinds of repeats not mentioned previously. SN _*all*_ is the sensitivity to all classes of repeats.

Coding regions, exons, may contain repeats known as integrated repeats. Thus, repeat-detection tools, including knowledge-based tools such as RepeatMasker, cannot avoid coding regions completely. Yet, successful repeat-detection tools should be able to exclude the majority of coding regions. Equation 7 defines the specificity, SP _*exon*_, which is the percentage of the coding nucleotides a tool is able to avoid.

(7) $$  {SP}_{exon} = 100 - 100 \times \frac{O}{E}  $$

*O* is the overlap between potential repeats detected by a tool and known exons (in bp), and *E* is the length of the known exons (in bp).

The percentage predicted (PP) is the percentage of nucleotides of a chromosome predicted to be repeats. The false positive length (FPL) is the total length of detections found in a random genome. The random genome is generated by a group of 6^*th*^ order Markov chains, each of which is trained on one chromosome. The corresponding random chromosome with the same length as the real chromosome is generated by the corresponding Markov chain. Because the random genome is similar in composition to the real genome, it may include valid repeats. Therefore, repeats located by RepeatMasker in the random genome are removed. The FPL is measured in bp. The ability of a tool to predict potential repeats has been used in previous studies [15, 16]. The potential repeats measure (PR) is the number of nucleotides that were predicted by a tool as repeats but were not detected by RepeatMasker. The PR content is measured in bp. To measure the time and the memory, all programs were executed on a supercomputer (a cluster). Each node of the cluster has two eight-core Intel Xeon E5-2680 processors at 2.7 GHz and 128 GB of RAM.

At this point, the evaluation measures have been defined. Next, I apply these measures to evaluate Red, RepeatScout, ReCon, and WindowMasker.

### Evaluations of the four tools

This study makes the following four main contributions: (i) the Red software; (ii) a rigorous evaluation of the current state of the art on the genomes of the following species: *Homo sapiens*, *Zea mays*, *Glycine Max*, *Drosophila melanogaster*, *Dictyostelium discoideum*, *Plasmodium falciparum*, and *Mycobacterium tuberculosis*; (iii) repeats found by Red in the genomes of the seven species (Additional files 5, 6, 7, 8, 9, 10, 11 and 12); and (iv) nearly 46,500 novel repetitive segments identified by Red in the human genome (Additional file 13). Table 1 displays comparisons of the performances of Red, RepeatScout, ReCon, and WindowMasker. Next, I elaborate on the advantages of Red over the other tools.

**Table 1:** Comparisons of the performances of RepeatScout, ReCon, WindowMasker, and Red. Repeats detected by RepeatMasker are considered the ground truth in this study

	SN _*te*_	SN _*tr*_	SN _*low*_	SN _*other*_	SN _*all*_	SP _*exon*_	PP	FPL	PR	Time	Memory
Tool	(%)	(%)	(%)	(%)	(%)	(%)	(%)	(bp)	(bp)	(sec)	(MB)
*Homo sapiens*– 3,099,750,718 bp											
RS	62.5	79.6	13.6	29.2	63.5	90.5	33.7	239474	9355324^a^	948,350	4701
Red	61.0	86.2	68.9	33.9	62.8	89.3	35.5	2657024	16414125^a^	5184	6775
WM	55.2	74.9	81.9	25.7	56.7	87.2	36.1	423707488	3109241^a^	14866	615
RC	55.0	75.2	11.0	11.5	56.2	95.4	29.2	137633	3575640^a^	898,844	14666
*Drosophila melanogaster*– 143,726,002 bp											
Red	90.0	59.4	43.3	83.0	84.1	94.0	23.2	312686	9401953	206	916
RS	86.3	24.3	1.9	71.8	74.4	98.0	18.4	0	4913141	79008	979
RC	86.7	18.2	1.8	80.0	74.0	99.0	17.6	0	4002422	13979	1513
WM	45.5	64.8	62.7	42.1	48.8	90.8	22.3	15150087	17118084	2869	325
*Zea mays*– 2,059,943,587 bp											
RS	96.7	55.5	25.8	89.9	96.3	–	80.0	44447	66587503	347082	7344
Red	93.3	58.1	31.9	88.7	93.0	–	78.8	6257	94687287	2731	6741
RC	91.6	33.5	12.9	88.8	91.1	–	74.3	20864	32450624	192223	3419
WM	82.3	63.7	40.1	86.6	82.1	–	67.2	36189699	33998795	7589	639
*Glycine max*– 973,344,380 bp											
RC	96.3	42.5	22.6	99.9	92.7	95.1	46.4	2144642	123719267	304490	8609
RS	92.5	39.6	19.1	94.4	89.0	92.0	43.6	2690420	110068092	134936	1516
Red	86.9	42.5	28.9	96.1	83.9	94.5	41.6	1794609	107704170	1653	1770
WM	68.1	83.4	83.6	3.5	68.9	95.4	44.4	170352943	186081334	13319	356
*Dictyostelium discoideum*– 34,121,699 bp											
Red	94.7	93.8	96.5	1.0	94.3	–	54.9	2378281	10582912	61	235
WM	35.1	92.7	95.1	4.8	84.7	–	53.0	14238455	10769264	20	2
RC	95.0	25.0	7.6	0.0	31.9	–	13.6	0	1883829	18317	957
RS	79.4	27.0	4.6	0.0	30.4	–	13.5	0	1969788	17476	925
*Plasmodium falciparum*– 23,264,338 bp											
WM	–	91.2	90.6	28.5	89.2	–	61.4	10902380	10246592	23	7
Red	–	87.6	84.2	90.7	87.2	–	51.8	972553	8129833	63	416
RS	–	43.9	9.9	40.1	39.4	–	15.3	36882	1797419	36194	918
RC	–	20.3	7.7	34.5	19.1	–	9.3	4827	1314829	7011	1052
*Mycobacterium tuberculosis*– 4,403,837 bp											
Red	–	88.7	81.0	–	88.5	–	44.0	160705	1914667	7	1
WM	–	63.6	33.3	–	63.0	–	17.5	672523	755248	2	2
RS	–	20.8	27.3	–	21.0	–	5.6	0	240852	331	640
RC	–	0.0	0.0	–	0.0	–	0.0	0	2089	69	852

SN _*te*_ is the sensitivity to all types of transposable elements. SN _*tr*_ is the sensitivity to tandem repeats including microsatellites and satellites. SN _*low*_ is the sensitivity to low complexity regions. SN _*other*_ is the sensitivity to repeats that are not transposons, tandem repeats, or low complexity regions. SN _*all*_ is the sensitivity to all types of repeats. SP _*exon*_ is the specificity to coding regions. PP stands for the percentage of the nucleotides of a chromosome predicted to be repeats. The False Positive Length (FPL) is the total length of repeats found in a synthetic random genome with the same length as the original genome; the synthetic genome is generated by a group of Markov chains of the 6^*th*^ order. Each chain is trained on one real chromosome. Repeats found in the synthetic genome by RepeatMasker were removed. Potential Repeats (PR) is the number of nucleotides that were found in the repeats predicted by a tool but not in the repeats located by RepeatMasker. The symbol “bp” stands for base pair. “MB” represents the unit megabyte. The ‘^a^’ next to the PR indicates that these repeats are confirmed novel repeats

#### Red is a totally independent system

ReCon depends on BLAST, Dialign, and RepeatMasker. Similarly, RepeatScout depends on RepeatMasker. Therefore, the users of ReCon and RepeatScout must install and learn how to use additional tools. In contrast, Red does not depend on other tools, simplifying its installation and use. WindowMasker does not depend on other tools as well; however, its users are required to write a script to process a whole genome. This extra step is not the case with Red.

#### Red has high sensitivities to both TE and TR

With regard to the sensitivity to TE, SN _*te*_, the performance of Red was the best or the second best tool on four genomes out of the five genomes that include TE (the genomes of the *Plasmodium falciparum* and the *Mycobacterium tuberculosis* do not include TE). ReCon and RepeatScout achieved high SN _*te*_, whereas WindowMasker had the lowest SN _*te*_. Regarding the sensitivity to TR, SN _*tr*_, Red had the highest or the second highest sensitivity on the seven genomes. WindowMasker achieved high SN _*tr*_, whereas ReCon and RepeatScout had the lowest SN _*tr*_. These results demonstrate Red’s capability of locating the two major classes of repeats. Additionally, these results show that the related tools perform well on either TE or TR, but not on both types.

#### Red performs consistently well on the tested genomes

Red was the most or the second most sensitive tool to all repeats including TE, TR, low complexity regions, and other types of repeats (SN _*all*_) in six genomes. Although the SN _*all*_ of Red on the *Glycine max* genome was high, it came third; however, it outperformed WindowMasker with a large margin (83.0 % vs. 68.9 %). RepeatScout performed well only on four genomes (*Homo sapiens*, *Zea mays*, *Glycine max*, and *Drosophila melanogaster*). Similarly, ReCon performed well only on three species (*Zea mays*, *Glycine max*, and *Drosophila melanogaster*). Likewise, WindowMasker achieved the best or the second best SN _*all*_ only on three species (*Dictyostelium discoideum*, *Plasmodium falciparum*, and *Mycobacterium tuberculosis*). In sum, Red performed consistently well on the seven genomes, while each of the other tools performed well on some of the genomes but not on all of the seven genomes.

#### Red is much faster than RepeatScout and ReCon

The difference in speed between Red and RepeatScout and ReCon is clear when it comes to large genomes. Red is faster than RepeatScout and ReCon by many folds. For example, RepeatScout processed the *Homo sapiens* genome in approximately 11 days; in contrast, Red processed the same genome in 87 minutes. Red analyzed the *Zea mays* and the *Glycine max* genomes 127 times and 82 times faster than RepeatScout. ReCon took slightly more than 10 days to process the *Homo sapiens* genome; in contrast, Red took 87 minutes. Red was 70 times and 184 times faster than ReCon on the genomes of the *Zea mays* and the *Glycine max*. Red is faster than WindowMasker by 3–14 times on the genomes of the *Homo sapiens*, the *Drosophila melanogaster*, the *Zea mays*, and the *Glycine max*. These results demonstrate that Red is the fastest tool on medium and large genomes.

#### Red has a much lower FPL than WindowMasker

In this study, it has been observed that RepeatScout and ReCon have low FPL and WindowMasker has high FPL. The FPL of Red was consistently lower than that of WindowMasker by many folds on the seven genomes. For example, the FPL of Red was tens to hundreds of times lower than the FPL of WindowMasker on the genomes of the *Homo sapiens*, the *Drosophila melanogaster*, the *Zea mays*, and the *Glycine max*. On the genomes of the *Dictyostelium discoideum*, the *Plasmodium falciparum*, and the *Mycobacterium tuberculosis*, which have unusual nucleotide compositions, the FPLs of Red were 6–11 times lower than those of WindowMasker. These results show that Red achieved high sensitivity, consistent performance, and high speed while maintaining low to moderate false positive rates.

#### Red has the ability to discover repeats in genomes that have unusual nucleotide compositions while maintaining moderate FPLs

The genome of the *Dictyostelium discoideum*, the social amoeba, is a unique genome. Repeats of this genome are unusual. Its TE are clustered, and its TR are very abundant and occur in stretches every 392 bp on average [46]. In addition, this genome has an unusual nucleotide composition; specifically its A-T content is 77.6 %. Red and ReCon achieved the highest *S* *N*_*te*_ (94.7 % and 95.0 %). Red and WindowMasker achieved the best *S* *N*_*tr*_ (93.8 % and 92.7 %). Overall, ReCon and RepeatScout had the lowest *S* *N*_*all*_ of 31.9 % and 30.4 %. In contrast, Red achieved the highest *S* *N*_*all*_ (94.3 %), followed by WindowMasker (84.7 %). Red’s FPL was 6 times less than that of WindowMasker. I conducted an additional set of evaluations on another unique species, *Plasmodium falciparum* (the parasite causing malaria in humans). This unique genome has the highest known A-T content of 80.6 % [47]. In addition, it does not include TE. WindowMasker achieved the highest *S* *N*_*tr*_ (91.2 %) followed by Red (87.6 %); however, Red’s FPL was 11 times less than that of WindowMasker. The overall sensitivities of WindowMasker and Red were comparable (89.2 % vs. 87.2 %). The overall sensitivities of RepeatScout and ReCon were 39.4 % and 19.1 %. These results confirm the ability of Red to discover repeats in genomes of unusual nucleotide compositions while maintaining moderate FPLs.

#### Red has the ability to discover repeats in bacterial genomes

*Mycobacterium tuberculosis* is the bacteria causing tuberculosis. Its genome is C-G rich (65.6 %). Repeats of the *Mycobacterium tuberculosis* genome include TR and low complexity regions mainly. Red outperformed all of the related tools with a large margin. Specifically, its overall sensitivity was 88.5 %, whereas the sensitivities of WindowMasker, RepeatScout, and ReCon were 63.0 %, 21.0 %, and 0.0 %. Further, the FPL of Red was 4 times lower than that of WindowMasker, the second best tool sensitivity wise. These figures demonstrate the successful application of Red to bacterial genomes.

#### Red has the ability to discover a large number of novel repetitive segments

Potential novel repeats are those located by a tool but not by RepeatMasker. One way to confirm these potential repeats is to count the number of their copies in the genome. Specifically, potential novel repeats are confirmed using the following procedure:

- Nucleotides of coding regions are removed.
- Nucleotides of known repeats are removed.
- Each of the remaining segments is aligned versus the whole genome by BLAST. Stringent BLAST parameters are used. These parameters ensure that the identity between a segment and a BLAST match is high (80 % for long, ≥50 bp, segments; 90 % for short, <50 bp, segments). Additionally, the chosen parameters guarantee that matches located by BLAST are true matches beyond any statistical doubt (See the Additional file 1).
- If the length of the alignment differs from the length of the query segment by more than 20 %, the match is removed.
- A segment is confirmed to be repetitive if 10 valid matches are found.
- If RepeatMasker masks 50 % or more of a segment, the segment is removed.

Based on this validation procedure, Red found 9,499 short (20–49 bp) confirmed novel repeats (CNR) and 36,906 long (≥50 bp) CNR totaling 46,405 segments in the human genome. Examples of Red’s CNR are shown in Table 2.

**Table 2:** Examples of the confirmed novel repeats found by Red in the genome of the *Homo sapiens* (hg38)

Location	Copy number	Length	Sequence
chr1:242110361–242110392	56058	31	ACATTCAAGTGATTCTCCTGCCTCAGCCTCA
chr2:119996761–119996800	51817	39	TCAATTGGCCGGGTGCGGTGGCTCACACCTGTAATCCCA
chr9:61982576–61982619	2292	43	TTGGGATTTCAGGCGTGAGCCACTGTGCCTGGCCAGCATTGCT
chrX:129964870–129964913	1344	43	TGTGTGTGGGTCTGTGTGTGAGAGAGAGAAAGAGAGAAACATG
chr16:55969556–55969604	1327	48	GTACATATATATACGTGTGTGTGTGTGTGTGTGTATATATATAAATTA
chr19:20397318–20397528	324	210	GCTTTGTTACAGTATTGGTTTCTGTCCACTATGAATTCTCTTATGTTTAT
			TGAAGTCTGAGGACCAGTTAAAAGCTTTGCCACATTCTTCACATTTGCAA
			GGTTTCTCTCCAGTATGAATTGTCTTATATTCACTTAGAGTTGAGGATGC
			AGTAAAGGCTTTGCCACATTCTTCACATTTGTAAGGTTTCTCTCCAGTAT
			GAGTTCTCCT
chr10:38932814–38933047	73	233	ACTAGGGTAGGTAATTTCATCTCAGTCTTATGCAGGTACCTTTTCTCAGG
			ATCTCAGGAATGCAGACTTCTCACACTTCTGTTCTTTTCCTGGCTGTGTT
			GGTGAGCTCAGTGATATTCCTCCATCACCTTCAAGAGCAGTTTTGTTTTG
			TTTTTCCTGTTTTCATACTCCCAGCATCAGGAGTGTTCTAGGTGTGTCAG
			TTTTTGTTACCTTCCCCTACATATTAAGTGGAA
chr18:79830653–79831758	15^a^	1105	TTCCCTGCGGACAGAGCCTTTGTCAGGAGGGTTCCCTGCAGACAGAGCCT
			TCGTCAGGAGGGTTCCCTGCAGACAGAGCCTTCGTCAGGAGGGTTCCCTG
			CGGACAGAGCCTTCGTCAGGAGGGTTCCCTGCGGACAGAGCCTTCGTCAG
			GAGGGTTCCCTGCATACAGAGCCTTCGTCAGGAGCGTTCTCTGCGGACAG
			AGCCTTCGTCAGGAGGGTTCCCTGCATACAGAGCCTTCGTCAGGAGGGTT
			CCCTGCGGACAGAGCCTTCGTCAGGAGGGTTCCCTGCGGACAGAGCCTTC
			GTCAGGAGGGTTCCCTGCGGACAGAGCCTTCGTCAGGAGGGTTCCCTGCG
			GACAGAGCCTTCGTCAGGAGGGTTCCCTGCGGACAGAGCCTTCGTCAGGA
			GGGTTCCCTGCGGACAGAGCCTTCGTCAGGAGGGTTCCCTGCGGACAGAG
			CCTTCGTCAGGAGGGTTCCCTGCGGACAGAGCCTTCGTCAGGAGGGTTCC
			CTGCGGACAGAGCCTTCGTCAGGAGGGTTCCCTGCGGACAGAGCCTTCGT
			CAGGAGGGTTCCCTGCGGACAGAGCCTTCGTCAGGAGGGTTCCCTGCGGA
			CAGAGCCTTCGTCAGGAGGGTTCCCTGCGGACAGAGCCTTCGTCAGGAGG
			GTTCCCTGCGGACAGAGCCTTCGTCAGGAGGGTTCCCTGCGGACAGAGCC
			TTCGTCAGGAGGGTTCCCTGCGGACAGAGCCTTCGTCAGGAGGGTTCCCT
			GCGGACAGAGCCTTCGTCAGGAGGGTTCCCTGCGGACAGAGCCTTCGTCA
			GGAGGGTTCCCTGCGGACAGAGCCTTCGTCAGGAGGGTTCCCTGCGGACA
			GAGCCTTCGTCAGGAGGGTTCCCTGCGGACAGAGCCTTCGTCAGGAGCGT
			GCCCTGCGTACAGAGCCTTCGTCAGGAGCGTGCCCTGCGTACAGAGCCTT
			CGTCAGGAGCGTGCCCTGCGTACAGAGCCTTCGTCAGGAGCGTGCCCTGC
			GGACAGAGCCTTCGTCAGGAGGGTTCCCTGCGGACAGAGCCTTCGTCAGG
			AGGGTTCCCTGCGGACAGAGCCTTCATCAGGAGGGTTCCCTGCGGACAGA
			GCCTT

The sequence chr18:79830653–79831758 has 15 overlapping copies, marked by ‘^a^’

Using the same validation procedure, the lengths of the CNR located by RepeatScout, ReCon, and WindowMasker are 9,355,324 bp; 3,575,640 bp; and 3,109,241 bp. In contrast, the length of the CNR identified by Red (16,414,125 bp) is almost double the length of those located by RepeatScout. These results demonstrate that Red discovered more novel repeats than RepeatScout, ReCon, and WindowMasker.

The CNR located by Red in the human genome include the majority of the nucleotides comprising the CNR identified by the other three tools. Specifically, Red’s CNR include 92.0 %, 86.9 %, and 77.3 % of those located by ReCon, RepeatScout, and WindowMasker. In contrast, the CNR identified by RepeatScout, ReCon, and WindowMasker include 49.7 %, 20.4 %, and 14.9 % only of the nucleotides comprising the CNR located by Red. These figures show that the majority of the CNR detected by the three tools were located by Red as well; however, it is not the other way around.

**Fig. 4:**
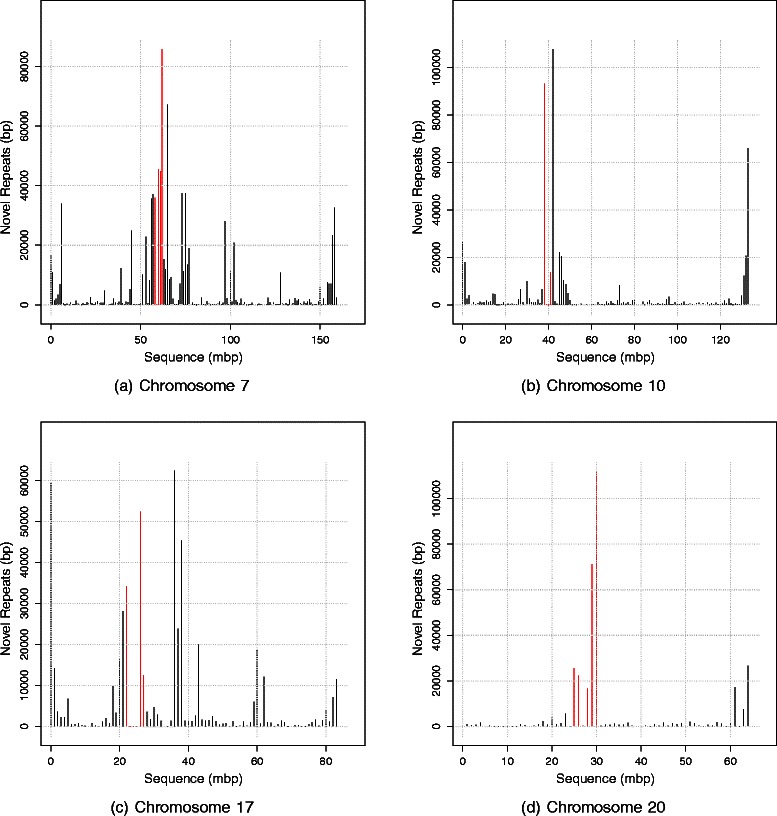
Distribution of the confirmed novel repeats (CNR) found by Red in four human chromosomes. The unit mbp stands for mega base pair. Each chromosome is divided into 1-mbp segments, which are plotted on the x-axis. The total size of the confirmed novel repeats detected by Red in each of the 1-mbp segments is displayed on the y-axis. Segments spanning the centromere of the chromosome are colored in red. (**a**) The distribution of the CNR in the human chromosome 7. (**b**) The distribution of the CNR in the human chromosome 10. (**c**) The distribution of the CNR in the human chromosome 17. (**d**) The distribution of the CNR in the human chromosome 20

It has been known that centromeres are rich with TR [48]. Assuming that the CNR are distributed uniformly throughout the chromosomes, 3.0 % of them are expected to be centromeric. However, 8.3 % of the CNR are centromeric. These figures indicate that the CNR are enriched in the centromeres (2.8 folds more than the expected value under a uniform distribution, p-value = 0, *χ* ^2^-square test). This finding supports the validity of the novel repeats detected by Red because centromeres are repeat-rich regions. Figure 4 shows the distribution of the CNR in four human chromosomes.

In addition, the CNR appear to be enriched at the peripheries, the telomeres, of the human chromosomes displayed in Fig. 4. To measure this enrichment at the genome level, I calculated the expected and the observed lengths of the CNR in the telomeres of each chromosome. For this purpose, a telomere is defined as the 1-mbp-long segments at the peripheries of a chromosome. Under a uniform distribution, the telomeric CNR are expected to make up about 1.6 % of the total length of the CNR. In contrast, the CNR detected by Red in the telomeres represent 10.3 % of the total length (6.6 folds more than the expected length, p-value = 0, *χ* ^2^-square test). Telomeres, similar to centromeres, are repeat-rich regions [48]. These results show that the CNR discovered by Red are enriched in the telomeres of the human chromosomes, supporting the validity of the novel repeats discovered by Red.

#### Red is capable of detecting repeats in unassembled genomes

To evaluate the performance of Red on an unassembled genome, I obtained the short reads of a *Drosophila melanogaster* genome from the *Drosophila* 1000 genomes project. To establish a baseline, I scanned the unassembled genome using Red trained on the assembled genome, Dm6. This model is referred to as Red _*dm*6_. The overall sensitivity, SN _*all*_, of Red _*dm*6_ to repeats located by RepeatMasker in the unassembled genome was 76.6 %. Repeats predicted by Red _*dm*6_ covered 32.3 % of the unassembled genome. Next, I trained Red on the short reads using adjusted parameters (see the Additional file 1). I call this model Red _*sr*_. In comparison to the baseline, using the unassembled genome for training improved the overall sensitivity considerably (92.2 % vs. 76.7 %) while maintaining similar percentages of predicted repeats (35.0 % vs. 32.3 %).

Because of the nature of the next generation sequencing technology, a non-repetitive short sequence may appear in many short reads. A successful tool for detecting repeats in unassembled genomes should be able to avoid such non-repetitive sequences. To ensure that Red is capable of avoiding these sequences, I scanned the assembled genome using Red _*sr*_. Recall that Red _*sr*_ was trained on the unassembled genome. Then I compared its performance to that of Red _*dm*6_ on the assembled genome. The percentages of repeats predicted by both models were comparable (21.4 % vs. 23.2 %). Further, the lengths of the potential repeats were similar (9,448,873 vs. 9,401,953). In addition, the SP _*exons*_ of the two models were comparable (94.2 % vs. 94.0 %). If repeats detected by Red _*sr*_ included non-repetitive sequences, the percentage of predicted repeats and the total length of potential repeats would be much higher and the the SP _*exons*_ would be much lower than those obtained by Red _*dm*6_. However, this was not the case because both models performed comparably when evaluated according to these three criteria. These results show that Red is capable of avoiding non-repetitive sequences when it is trained on unassembled genomes.

Next, I compared the overall sensitivities and the false positive lengths of the two models on the assembled genome to study the effects of the quality of the assembly on these two measures. As expected, the *S* *N*_*all*_ of Red _*sr*_ was lower and its FPL was higher than those obtained by Red _*dm*6_ (*S* *N*_*all*_: 68.8 % vs. 84.1 %, FPL: 3,599,391 vs. 312,686). However, Red _*sr*_ outperformed WindowMasker, which is the fourth performing tool that was trained and evaluated on the assembled genome (SN _*all*_: 68.8 % vs. 48.8 %, FPL: 3,599,391 vs. 15,150,087).

In sum, Red _*sr*_ has excellent performance on the unassembled genome. Evaluating Red _*sr*_ on the assembled genome showed its ability to avoid non-repetitive sequences that appear tens of times in the short reads. As expected, Red _*sr*_ under performed Red _*dm*6_ on the assembled genome. However, Red _*sr*_ outperformed WindowMasker that was trained on the assembled genome. These results demonstrate the successful application of Red to unassembled genomes.

## Discussion

In this section, I start with studying two confirmed novel repeats. After that, I discuss the specificity to coding regions. Next, the advantages of using Red as a repeat-masking tool are listed. Afterward, I discuss the related problem of classifying repeats. Then I highlight directions for improving Red. Finally, I conclude.

### Case study 1 - a centromeric confirmed novel repeat

I investigated one of the confirmed novel repeats located in the centromere of the human chromosome 10. The sequence of the novel repeat, chr10:38932814–38933047, is shown in Table 2. This sequence is 233 bp long. BLAST located 73 copies of this sequence. 61 copies are mapped to several human chromosomes, whereas the remaining 12 copies are present in random fragments of the genome. I studied the 61 copies to know whether or not they occur in the centromeres of the other chromosomes. Interestingly, I found 41 (67.0 %) out of the 61 copies to be present in the centromeres of 11 human chromosomes. Table 3 shows the locations of the 61 copies. These results suggest that these sequences are associated with the centromeres of the human chromosomes. In addition, 12 copies are present in fragments without known locations in the genome, suggesting that these fragments are likely to be centromeric. Such information may improve the assembly of the human genome.

**Table 3:** Copies of the 233-bp-long centromeric novel repeat: chr10:38932814–38933047. There are 73 copies of this novel repeat found throughout the human genome. The locations of 61 of these copies are known, whereas 12 of them are mapped to random segments of the genome. Out of the 61, 41 (67.0 %) copies are located in or within 1 mbp from the centromeres of several human chromosomes

BLAST Hist	Identity (%)	Centromeric?	BLAST Hist	Identity (%)	Centromeric?
chr1:125097011–125096780	94.9	yes	chr9:65753438–65753671	94.9	no
chr1:125143875–125144108	92.7	yes	chr10:42109674–42109443	95.3	yes^a^
chr1:143279916–143279685	92.7	no	chr10:42120842–42120611	94.9	yes^a^
chr1:143535471–143535704	93.6	no	chr15:19934467–19934700	94.9	yes
chr2:90335400–90335172	94.4	yes^a^	chr16:32106608–32106841	94.4	no
chr2:91468090–91468320	94.8	yes	chr16:32821244–32821013	94.4	no
chr2:91800041–91800275	94.4	yes	chr16:33049905–33050138	94.4	no
chr2:92052731–92052499	94.4	yes	chr16:34041061–34041294	95.3	yes^a^
chr2:92076458–92076691	95.3	yes	chr16:34460177–34459946	91.9	yes^a^
chr2:94282644–94282411	94.5	yes	chr16:34497844–34497619	94.3	yes^a^
chr2:132008526–132008295	94.9	no	chr16:34510019–34509788	94.0	yes^a^
chr2:132047806–132047581	92.7	no	chr16:34653787–34654020	93.1	yes^a^
chr7:53130967–53130730	91.2	no	chr16:34692352–34692578	93.4	yes^a^
chr7:57878379–57878148	94.4	yes^a^	chr16:34792862–34793095	93.1	yes^a^
chr7:58101480–58101713	94.4	yes	chr16:34838562–34838795	94.4	yes^a^
chr7:60906762–60906995	94.9	yes	chr16:34850733–34850960	94.3	yes^a^
chr7:60982800–60983033	94.9	yes	chr16:34888413–34888646	91.9	yes^a^
chr7:61076250–61076019	94.9	yes	chr16:34943100–34943333	94.9	yes^a^
chr7:61583132–61582901	94.9	yes	chr16:36163632–36163865	93.1	yes
chr7:62318164–62317933	94.4	yes	chr16:36222879–36223112	91.9	yes
chr7:62398128–62398361	94.4	yes	chr16:46425718–46425487	94.4	no
chr7:62434242–62434475	94.9	yes	chr16:46436413–46436188	92.3	no
chr7:65114062–65114290	92.3	no	chr17:26756876–26757109	95.7	yes
chr7:65519041–65518815	92.3	no	chr17:26953504–26953737	97.4	yes
chr7:65581931–65581705	92.3	no	chr18:15163745–15163514	95.7	yes
chr9:40660714–40660483	94.9	no	chr18:15207260–15207029	94.4	yes
chr9:43290928–43290698	95.3	yes	chr21:8585703 -8585935	94.0	no
chr9:43313883–43314115	95.3	yes	chr21:10618400–10618633	94.4	yes
chr9:63460122–63459891	94.9	no	chr22:10562609–10562841	94.0	no
chr9:64784259–64784028	94.9	no	chr22:16255856–16256089	95.7	yes
chr9:65268071–65267840	94.9	no			

Copies within 1 mbp from the centromeres are marked by ‘^a^’

### Case study 2 - a telomeric confirmed novel repeat

Table 2 displays a 1105-bp long novel minisatellite (chr18:79830653–79831758). This sequence is located at the telomere of the human chromosome 18. The minisatellite consists of 35 almost-identical copies of the following motif: TTCCCTGCGGACAGAGCCTTTGTCAGGAGGG.

### The specificity to coding regions

It is important to evaluate the specificity, SP _*exon*_, of a repeat-detection tool in particular for studies focusing on coding regions. The SP _*exon*_ was calculated on the genomes of the *Homo sapiens* and the *Drosophila melanogaster* because the coding regions of these genomes are well annotated. Repeats located by RepeatMasker included 10.3 % of the nucleotides comprising known exons in the human genome, i.e. the SP _*exon*_ of RepeatMasker was 89.7 %. The SP _*exon*_ of WindowMasker was lower than that of RepeatMasker (87.2 % vs. 89.7 %), whereas the SP _*exon*_ of Red was comparable to that of RepeatMasker (89.3 % vs. 89.7 %). RepeatScout had a slightly higher SP _*exon*_ than that of RepeatMasker (90.5 % vs. 89.7 %). The SP _*exon*_ of ReCon was much higher than that of RepeatMasker (95.4 % vs. 89.7 %). On the genome of the *Drosophila melanogaster*, the SP _*exon*_ of ReCon and RepeatScout were higher than that of RepeatMasker (99.0 % and 98.0 % vs. 96.5 %). Red’s SP _*exon*_ was lower than that of RepeatMasker (94.0 % vs. 96.5 %), whereas the SP _*exon*_ of WindowMasker was much lower than the SP _*exon*_ of RepeatMasker (90.8 % vs. 96.5 %). These numbers can be utilized differently according to the domain of the application. When repeats are the focus of a study, the SP _*exon*_ is of minor importance as long as it is within a reasonable range. When coding regions are the focus of a study, the higher the SP _*exon*_, the better. In practice, a performance similar to that of RepeatMasker is the best performance that can be hoped for because RepeatMasker uses a library of manually annotated repeats. Red’s SP _*exon*_ on the human genome was comparable to that of RepeatMasker. The SP _*exon*_ of Red on the genome of the *Drosophila melanogaster* was 2.5 % lower than that of RepeatMasker. These results show that Red has the ability to avoid a large percentage of the nucleotides comprising coding regions, making Red a well-suited tool for studies focusing on repeats or on coding regions.

### The specificity to coding regions of duplicated human genes

Red, RepeatScout, ReCon and WindowMasker were evaluated on 3543 duplicated human genes obtained from the Duplicated Genes Database [49]. These genes were divided into three groups according to their copy number. The groups are the 2–4 group, the 5–9 group, and the 10-or-more group. The SP _*exon*_ of each tool was calculated on each group. Additionally, the SP _*exon*_ of RepeatMasker was evaluated on each group to establish a baseline. Table 4 provides the results. The SP _*exon*_ of ReCon was consistently higher than that of RepeatMasker on each of the three groups; however, ReCon had the lowest overall sensitivity. The figures of WindowMasker on the 2–4 group and the 10-or-more group were lower than those of RepeatMasker. WindowMasker and RepeatMasker had comparable SP _*exon*_ on the 4–9 group. Red and RepeatScout, the two most sensitive tools, had similar performances to that of RepeatMasker (89.4 % and 89.9 % vs. 89.4 %) on the 2–4 group. On the 5–9 group, Red achieved a comparable SP _*exon*_ to that of RepeatMasker (87.8 % vs. 88.6 %), whereas RepeatScout had a much lower SP _*exon*_ than that of RepeatMasker (75.2 % vs. 88.6 %). On the 10-or-more group, Red’s SP _*exon*_ was lower than that of RepeatMasker (68.5 % vs. 84.3 %). However, the SP _*exon*_ of RepeatScout was much lower than those of Red and RepeatMasker (53.1 % vs. 68.5 % and 84.3 %). These results demonstrate that Red is capable of avoiding 88 %–89 % of the coding nucleotides making up genes that have 2–9 copies. Additionally, Red outperformed RepeatScout, which achieved a comparable best performance on the human genome, by a large margin when the number of gene copies is 5 or more.

**Table 4:** The specificity to nucleotides comprising duplicated human genes. Duplicated genes were divided into the following three groups according to their copy numbers: the 2–4 group, the 5–9 group, and the 10-or-more group. SP _*exon*_ is the percentage of the nucleotides of the genes in a group that are excluded by a tool

Gene Copy Number	2–4	5–9	≥10
Length (bp)	2,582,680	447,130	708,127
	*S* *P*_*exon*_ (%)		
ReCon	95.4	95.1	90.4
RepeatScout	89.9	75.2^a^	53.1^a^
Red	89.4	87.8	68.5
WindowMasker	87.6^a^	88.4	80.6
RepeatMasker	89.4	88.6	84.3

The lowest SP _*exon*_ on a gene group is marked by ‘^a^’

### The specificity to coding regions in polyploid genomes

The *Glycine max*, soybean, genome is tetraploid. It has been estimated that 75 % of its genes are “present in multiple copies” [50]. Therefore, it is important to evaluate the SP _*exon*_ of the four tools on this genome. The best available performance is due to RepeatMasker using RepBase. Repeats located by RepeatMasker included 4.0 % of the nucleotides comprising the coding regions of the *Glycine max* genome, i.e. the SP _*exon*_ of RepeatMasker was 96.0 %. The SP _*exon*_ of WindowMasker, ReCon, and Red were comparable to that of RepeatMasker (95.4 %, 95.1 %, 94.5 % vs. 96.0 %), whereas the SP _*exon*_ of RepeatScout was lower than that of RepeatMasker (92.0 % vs. 96.0 %). Red’s high specificity is due to the background model. The background model is a 6 ^*th*^ order Markov chain trained on the *Glycine max* genome. A 6 ^*th*^ order Markov chain is able to capture the polyploidy of this genome, estimating the expected count of a word accurately. Recall that the observed count of a word is adjusted by extracting its expected count. Therefore, the adjusted count of a word occurring in a non-repetitive region or a coding region is 0 on average. For example, consider a gene that has 4 copies in a tetraploid genome, i.e. 4 ohnologous genes. For simplicity, assume that the gene consists of unique words, and the four nucleotides – A, C, G, and T – are present in the genome in equal percentages. The observed count of a word present in this gene is 4. Similarly, the average expected count calculated by the background model of this word is 4. Thus, the adjusted count is 0 = 4 (the observed count) - 4 (the expected count). In the case of polypoid genomes, the observed count is adjusted by subtracting the polyploidy captured by the trained Markov chain. To further avoid non-repetitive and coding regions, a word is considered repetitive if its adjusted count is at least three. In sum, the background model utilized in Red is capable of capturing the polyploidy of the genome of interest, enabling Red to exclude the majority of coding nucleotides.

### Red as a repeat-masking tool

At the current stage, Red can be utilized as a repeat-masking tool. Given its consistent performance on seven genomes including those with unusual nucleotide compositions, Red is expected to mask repeats in newly sequenced genomes accurately. Precise exclusion of repeats improves the annotation of genomes, leading to better delineation of coding regions and regulatory modules. In addition, it has been reported that masking TR improves the performance of alignment tools [11]. Because Red is capable of locating TR de-novo, using Red for masking genomes should improve the quality of the alignments. Further, Red discovered novel repeats totaling 16,574,339 bp in the *Homo sapiens* genome. Excluding these confirmed novel repeats from search databases should reduce the search time dramatically.

### Classifying repeats

The problem of classifying repeats into families is the most challenging problem in the process of repeats annotation. The user wishes to collect copies of the same element in one group. This task is difficult because repeats present in a genome can be approximate copies of each other. Further, repeats can be present as solo repeats, i.e. they have delineating features such as long terminal repeats, without a sequence in between. Moreover, some of the copies may be partial copies. Finally, repeats can be nested within each other at several levels. Therefore, a simplistic approach is unlikely to produce good results. Given the difficulty of the problem and the sophistication required for building a computational tool for this purpose, I did not attempt to merge it with the problem at hand concerning repeats detection.

### Future improvements

Although Red is rapid and its memory requirements are available on personal computers, further improvements can be introduced. Specifically, I plan to explore means to further reduce the processing time and the memory requirement in future releases. At another level, I will focus on increasing the sensitivity of Red to the known human repeats. Although Red’s overall sensitivity ranges from 83.9 % to 94.3 % on six genomes, its sensitivity to the human repeats is 62.8 %. Because Red is one of the most sensitive tools on the human genome, I will investigate de-novo methods to improve the sensitivity on complex genomes such as the human genome.

## Conclusion

The genomes of thousands of species will be sequenced soon. Repeats are a major component of almost all genomes. Consequently, repeat-detection tools are needed to help annotate the newly sequenced genomes. Because repeats are species specific, repeats of newly sequenced genomes are unknown. Thus, de-novo repeat-detection tools are essential in the annotation process. However, many of the currently available de-novo tools cannot process an entire genome. Furthermore, tools that function on the genomic scale suffer from five limitations. Available tools (i) can be very slow; (ii) may have high false positive rates; (iii) are too difficult for average users, (iv) tend to be sensitive to either TR or TE, but not to both types of repeats; or (v) perform well on some genomes but not on others. The goal of my research is to invent a tool that can overcome these limitations. To this end, I designed and developed Red using Signal Processing and Machine Learning as well as a novel data structure I designed to handle long DNA sequences efficiently. To the best of my knowledge, *Red is the first repeat-detection tool that has the ability to label its own training data and to train itself automatically on each genome.* My evaluation of Red and the three related tools demonstrated that Red is a rapid, accurate, consistent and easy to use tool for detecting repeats in assembled and unassembled genomes. Additionally, Red is capable of discovering novel repeats; for example, Red discovered more than 46,000 novel repetitive segments in the human genome. These results, in addition to the novel methodology implemented in Red, represent a true advancement in the processes of repeat detection and genome annotation.

## Availability and requirements

The C++ source code and the binaries for Unix 64-bit and Mac 64-bit are available as Additional files 2,3, and 4. The most updated version is available at the project home page.

**Project name:** Red**Project home page:** http://toolsmith.ens.utulsa.edu **Operating systems:** Unix, Linux, and Mac OS X**Programming language:** C++**License:** The code provided by the author, National Center for Biotechnology Information (NCBI), National Library of Medicine, is a work of the U.S. Government and is not subject to copyright protection in the United States.

## Additional files

Additional file 1
**Supplementary methods.** This file includes more information about the methodology of Red and the data used in this study. Specifically, it comprises the following: (i) the run time analysis of Red showing that the time required by Red is linear with respect to the genome size; (ii) a discussion of the default values of Red’s parameters; (iii) the details of executing the related tools; and (iv) the sources of the data used in this study.

Additional file 2
**Supplementary data set 1 — Source code.** The command “tar -xzf file” results in a directory that contains the C++ source code of Red and instructions on how to compile the source code.

Additional file 3
**Supplementary data set 2 — Binary for Unix 64-bit.** The command “tar -xzf file” results in a directory that contains Red’s binary executable file compiled on a Unix 64-bit operating system and the manual.

Additional file 4
**Supplementary data set 3 — Binary for Mac 64-bit.** The command “tar -xzf file” results in a directory that contains Red’s binary executable file compiled on a Mac 64-bit operating system and the manual.

Additional file 5
**Supplementary data set 4 — Red’s repeats of the** ***Homo sapiens*** **genome.** The command “tar -xzf file” results in a directory that contains the locations of repeats found by Red in the genome of the *Homo sapiens*.

Additional file 6
**Supplementary data set 5 — Red’s repeats of the** ***Drosophila melanogaster*** **genome.** The command “tar -xzf file” results in a directory that contains the locations of repeats found by Red in the genome of the *Drosophila melanogaster*.

Additional file 7
**Supplementary data set 6 — Red’s repeats of the** ***Drosophila melanogaster*** **unassembled genome.** The command “tar -xzf file” results in a directory that contains the locations of repeats found by Red in an unassembled genome of the *Drosophila melanogaster*. In addition, the directory includes the short reads comprising the genome. This file can be download using the following link: https://drive.google.com/file/d/0B8O-qv7WO7L2LUViT1ZKWTNTUlE/edit?usp=drive_web

Additional file 8
**Supplementary data set 7 — Red’s repeats of the** ***Zea mays*** **genome.** The command “tar -xzf file” results in a directory that contains the locations of repeats found by Red in the genome of the *Zea mays*.

Additional file 9
**Supplementary data set 8 — Red’s repeats of the** ***Glycine max*** **genome.** The command “tar -xzf file” results in a directory that contains the locations of repeats found by Red in the genome of the *Glycine max*.

Additional file 10
**Supplementary data set 9 — Red’s repeats of the** ***Plasmodium falciparum*** **genome.** The command “tar -xzf file” results in a directory that contains the locations of repeats found by Red in the genome of the *Plasmodium falciparum*.

Additional file 11
**Supplementary data set 10 — Red’s repeats of the** ***Dictyostelium discoideum*** **genome.** The command “tar -xzf file” results in a directory that contains the locations of repeats found by Red in the genome of the *Dictyostelium discoideum*.

Additional file 12
**Supplementary data set 11 — Red’s repeats of the** ***Mycobacterium tuberculosis*** **genome.** The command “tar -xzf file” results in a directory that contains the locations of repeats found by Red in the genome of the *Mycobacterium tuberculosis*.

Additional file 13
**Supplementary data set 12 — Red’s novel repeats of the** ***Homo sapiens*** **genome.** The command “tar -xzf file” results in a directory that contains novel repeats found by Red in the *Homo sapiens* genome. Files that have the extension “.short” contain the short (20–49 bp) CNR. Files that have the extension “.long” contain the long (≥50 bp) CNR. Each file lists the location, the number of copies in the genome, the length, and the sequence of each novel segment. Files ending with the extension “.blast” include the corresponding matches found by BLAST for the long and the short CNR. This file can be download using the following link: https://drive.google.com/file/d/0B8O-qv7WO7L2ZGpPRUhlRUNodlE/edit?usp=drive_web.

## Abbreviations

LTR: Long terminal repeat
MS: Microsatellites
TE: Transposable elements
TR: Tandem repeats
HMM: Hidden Markov model
SN: Sensitivity
SP: Specificity
PP: Percentage predicted
FPL: False positive length
PR: Potential repeats
CNR: Confirmed novel repeats
