# Supplementary material for: Red: an intelligent, rapid, accurate tool for detecting repeats de-novo on the genomic scale
Source: BMC Bioinformatics. 2015 Jul 24;16:227. doi: 10.1186/s12859-015-0654-5 (PMC4513396; doi:10.1186/s12859-015-0654-5)
Supplement: Additional file 1 — Supplementary methods. This file includes more information about the methodology of Red and the data used in this study. Specifically, it comprises the following: (i) the run time analysis of Red showing that the time required by Red is linear with respect to the genome size; (ii) a discussion of the default values of Red’s parameters; (iii) the details of executing the related tools; and (iv) the sources of the data used in this study. [file 12859_2015_654_MOESM1_ESM.pdf]

Supplementary to
Red: an intelligent, rapid, accurate tool for detecting repeats de-novo on the genomic scale

Hani Z Girgis*[1]

[1] Computational Biology Branch, National Center for Biotechnology Information, National Library of Medicine, National Institutes of Health, 8600 Rockville Pike, Bethesda, MD 20894
[2] Tandy School of Computer Science, University of Tulsa, 800 South Tucker Drive, Tulsa, OK 74104

Email: Hani Z Girgis*- girgishz@mail.nih.gov;

*Corresponding author

Supplementary Methods

In this section, I discuss the following: (i) how the labeling module delineates candidate repetitive regions and potential non-repetitive regions, (ii) the run-time analysis of Red, (iii) the default values of Red's parameters, (iv) Red's parameters on the unassembled *Drosophila melanogaster* genome, (v) how the related tools were executed, and (vi) the source of the data used for evaluating the performance of Red and the related tools.

Delineation of candidates and potential non-repetitive regions

Once the local maxima have been found by the labeling module, the boundaries of the candidate regions are determined. Locating candidate repetitive regions involves the interleaving non-repetitive regions. A region is considered non-repetitive (repetitive) if the percentage of low ($\leq$ a user-specified threshold) scores in this region is greater (less) than the expected percentage assuming a uniform background distribution. The expected percentage is the percentage of the scores that are $\leq$ the score of the threshold in the genome. For example, suppose that 55% of the scores of a genome are ≤ 2. If these low scores were distributed uniformly, then one would expect the percentage of low scores in a random segment of this genome to be about 55%. However, due to the fact that non-repetitive regions include a high percentage of low scores, the observed percentage should be greater than 55%. In the current implementation of Red, if the expected percentage is low, it is increased to 52.5%.

Properties of repetitive regions are utilized for reducing the number of false maxima. The original scores

in the small region flanking a local maximum are examined to ensure that this maximum is in a repetitive region; the percentage of the low scores in the targeted region must be lower than the expected percentage. The targeted region has the same size as the Gaussian mask.

The presence of local maxima and of high scores is characteristic of repeats, whereas non-repetitive regions consist mainly of low scores. Two definitions are required. A separator and a core illustrate how the boundaries of repetitive regions are determined. A separator is defined as a non-repetitive region located between two consecutive local maxima; consequently, a separator does not include any local maximum. A core is defined as a repetitive region including at least one local maximum, and it is bounded by two separators. To begin the delineation of repetitive regions, the separators are identified and, in turn, the cores. Then, the boundaries of each core are adjusted.

The boundaries of a core are expanded or eroded in two stages. The first stage is a step-by-step expansion. During this stage, the small region adjacent to the start or the end of the core is added to the core if this region is a repetitive region. The size of this region, i.e. the step, is half the width of the Gaussian mask. This stage is repeated until a non-repetitive region is encountered. In the second stage, the start and the end of the core are further expanded or eroded nucleotide by nucleotide. The one-by-one expansion is executed if the score adjacent to the core is greater than that of the threshold. In this case, the boundaries of the core are adjusted to include this score. Expanding the core is repeated until a score that is less than or equal to the score of the threshold is encountered. The one-by-one erosion is executed if the original score at the start or the end of the core is less than or equal to that of the threshold. Then the boundaries of the core are adjusted to exclude this score. Eroding the core is repeated until a score that is greater than that of the threshold is encountered. During the second stage, the start or the end of the core is either expanded or eroded; however, it is not subject to the two operations combined.

Run time analysis

In order to determine the total run time of Red, I calculate the run time depending on the implementation, i.e. the code, of each of the four modules. To start, consider a genome of length n, a background Markov chain of the oth order, a mask of size m, and an HMM with s states. The following is an analysis of the time required by each module.

- **The scoring module:** This module takes n to count the k-mers, $o \times n$ to train the background Markov chain, and a constant time c to adjust the counts of k-mers. Thus, the total time taken by the scoring module is $(o + 1) \times n + c$.

- **The labeling module:** It takes n to score a sequence, $m \times n$ to smooth the scores, n to calculate the first derivative, n to calculate the second derivative, n to find the local maxima, at most n to find the separators, and at most n to expand the candidate regions. Therefore, the total time required by the labeling module is $(6 + m) \times n$.
- **The training module:** The module takes n to score a sequence, n to calculate the states using a logarithmic function of the scores, at most n to calculate the prior probabilities, and n to calculate the transition probabilities. The total time required by the training module is $4 \times n$.
- **The scanning module:** It takes n to score a sequence, n to calculate the states, and $s \times n$ to calculate the optimal series of states according to Viterbi's algorithm. The total time for the scanning module is $(2 + s) \times n$.

In sum, the total run time of Red is $(13 + o + s + m) \times n + c$. Because c, o, s, and m are constants, the upper bound of the run time is *O(n)*, i.e. linear with respect to the length of the genome.

System defaults

The system allows the user to specify the value of five parameters. However, an average user is likely to use Red with the default settings. Therefore, the default values of the parameters are important. Red has the following parameters.

Word length

The default value is equal to the floor of the logarithmic (base 4) value of the effective length of the genome. The effective length of a genome is the number of all nucleotides except the ones labeled as "N." The minimum default value is 12, and the maximum default value is 15. The user can adjust the length of the word using the "-len" parameter.

Order of the background Markov chain

The default value equals $\lfloor \text{word length} \div 2 \rfloor - 1$. On one hand, if the order is too small, the background Markov chain may not be realistic. On the other hand, if the order is too large, the background Markov chain may memorize the genome. Therefore, a Markov chain that has an order of roughly half of the word length provides practical estimation of the expected count of a word in a genome with a similar composition of short words. The user can adjust the order using the "-ord" parameter.

Half width of the Gaussian mask

The value of this parameter controls the width of the mask. The size of the mask is twice the value of this parameter. The standard deviation of the Gaussian distribution equals the value of this parameter divided by 3.5 because almost all samples fall within 3.5 standard deviations on each side of the mean. There are two default values: 20 and 40. If the G-C content is greater than 67% or less than 33%, the default value is 40. Otherwise, the default value is 20. The user can adjust the half width using the "-gau" parameter.

Score of the threshold of low scores in non-repetitive regions

The default score of the threshold of low scores is 2. A score of 2 indicates that the observed count of a k-mer in the genome is greater than the expected count by 2. Non-repetitive regions including duplicated segments consist mainly of less abundant k-mers. Therefore, a threshold of 2 suits the definition of non-repetitive regions. The user can adjust the score of the threshold using the "-thr" parameter.

Minimum observed copy number of a word

For a word having observed copy number less than or equal to the value of this parameter, the adjusted count is zero. The default value of this parameter is 2 to avoid duplicated segments. This parameter is very important for processing unassembled genomes (see the next section). The user can adjust the value of this parameter using the "-min" parameter.

Red's parameters on the unassembled Drosophila melanogaster genome

I obtained the short reads of a *Drosophila melanogaster* genome from the *Drosophila* 1000 genomes project. The average coverage of each genome in the project is 10 times. I used the forward reads only in this test. This data set consists of 21,806,206 76-bp-long sequences totaling 1,657,271,656 bp. The parameters of Red were adjusted to process the unassembled genome. Specifically, the word length was 16 and the minimum number of the observed words was 30 (3× the average coverage). I set the default minimum number of the observed words in an assembled genome to 3 to avoid duplicated segments. In the case of an unassembled genome, there are 30 (3× the average coverage) short reads, on average, covering each nucleotide of a segment that occurs in the genome 3 times.

Executing other tools

To evaluate the performance of Red, I compared it to RepeatScout, ReCon, and WindowMasker. In this study, I tried to evaluate other de-novo tools. However, RepeatScout, ReCon, and WindowMasker have been the only tools capable of processing the human genome or at least one human chromosome. The rest of the tools I tested have been unsuccessful in accomplishing the same task. The sensitivities of the tools were assessed with respect to the repeats located by RepeatMasker.

RepeatMasker

The ground truth consisted of repeats detected by RepeatMasker. RepeatMasker searches a sequence for instances of repeats in RepBase [1], which is a library of manually annotated repeats. BLAST was the search engine. Default values were used for all of the parameters. The following command was used for locating instances of known repeats:

RepeatMasker sequenceFile -s -species 'Species name' -dir outputDirectory

RepeatScout

RepeatScout was used with the default parameters. The program comes with some scripts to filter out simple repeats and low complexity regions. None of these filters was applied because the task at hand is the identification of all repeats, rather than the identification of transposable elements only. First the length of the word, k, is determined using the following equation that is suggested by the the inventors of RepeatScout:

k = round(log(chromosomeLength) / log(4)) + 1

Next, RepeatScout processes a chromosome to generate a library of repeats using the following commands:

> build_lmer_table -l k -sequence sequenceFile -freq tableFile

> RepeatScout -sequence sequenceFile -output libraryFile -freq tableFile -l k

Then RepeatMasker utilizes this library for locating repeats in the same sequence file using the following command:

> RepeatMasker sequenceFile -nolow -no_is -lib libraryFile -dir outDir

ReCon

I used ReCon with the default settings. Hundreds of 20-kbp-long segments were sampled from all chromosomes, or contigs, of a genome. The total number of the samples collected from the genomes of the *Glycine max*, the *Zea mays*, the *Drosophila melanogaster*, the *Homo sapiens*, the *Dictyostelium discoideum*, the *Plasmodium falciparum*, and the *Mycobacterium tuberculosis* are 12970, 4443, 3633, 1703, 1476, 1156, and 220. BLAST [2] processes the samples to produce all-vs-all alignments using the following commands:

```
> makeblastdb -in samplesFile -dbtype nucl -out blastDbFile
> blastn -db blastDbFile -query samplesFile -outfmt 6 -out blastOutFile
```

Next, the output of BLAST is further processed as recommended by the inventors of ReCon; a complete match between a query sequence and itself is removed. Each sequence is used as a query sequence and as a subject sequence. Thus, there are two identical pairs of matches; one of them is removed. The processed file is called 'mspFile.' A file including the names of the sequences is called 'namesFile.' ReCon processes the 'mspFile' using the following command:

```
> recon.pl namesFile mspFile 1
```

The output of ReCon is processed by Dialign [3] to construct a library of consensus sequences. Dialign aligns the longest 10 members of each family to produce a consensus sequence using a "majority vote." The longest 10 members are written to a file called 'familyFile.' The consensus sequence representing a family is generated using the following command:

```
> dialign2-2 -n -fa familyFile
```

The consensus sequences are written to a file called 'libraryFile.' Finally, RepeatMasker searches for repeats in a chromosome using the following command:

```
> RepeatMasker sequenceFile -nolow -no_is -lib libraryFile -dir outDir
```

WindowMasker

WindowMasker was used with the default settings. Unlike RepeatScout and ReCon, the output of WindowMasker is the repeats themselves not a library of consensus sequences. WindowMasker processes a genome in two stages. At the first stage, it counts k-mers occurring in a genome using the following command:

> windowmasker -mk_counts -fa_list true -in chromListFile -mem 5000 -out countFile

In the second stage, WindowMasker searches for repeats in a particular chromosome. The following command executes this task:

> windowmasker -ustat countFile -in file -out wmFile

BLAST

BLAST is used for validating the novel repetitive sequences discovered in the human genome. First, a database of the human genome is made using the following command:

> makeblastdb -in hg38.fa -dbtype nucl -out hg.db -title "Hg38"

Next, for segments of lengths of 20-49 bp, BLAST is executed with the following command:

> blastn -task blastn-short -db hg.db -query short.fa -evalue 1e-4 -perc_identity 90.0 -outfmt 6 -out short.txt

Finally, for segments that have at least 50 bp, BLAST is executed with the following command:

> blastn -db hg.db -query long.fa -evalue 1e-4 -perc_identity 80.0 -outfmt 6 -out long.blast

Data

Red and the related tools described above were evaluated on the sequences of seven genomes. The following list provides the version and the source of each genome:

- *Zea Mays* (AGPv3.21):
  http://ensembl.gramene.org/Zea_mays/Info/Index

- *Homo sapiens* (HG38):
  https://www.ncbi.nlm.nih.gov/Ftp/
  http://hgdownload.soe.ucsc.edu/goldenPath/hg38/bigZips/

- *Drosophila melanogaster* (DM6):
  http://hgdownload.soe.ucsc.edu/downloads.html#fruitfly

- *Drosophila melanogaster* (SRR350908 - unassembled short reads):
  http://www.ncbi.nlm.nih.gov/sra/?term=SRR350908

- *Plasmodium falciparum* (3D7):

  http://www.sanger.ac.uk/resources/downloads/protozoa

- *Dictyostelium discoideum*:

  http://dictybase.org/Downloads/

- *Mycobacterium tuberculosis* (CDC1551)

  http://genome.tbdb.org

- *Glycine Max* (Gmax_109)

  http://www.plantgdb.org/XGDB/phplib/download.php?GDB=Gm

RepeatMasker's repeats of the human genome were obtained from the following site:

http://www.repeatmasker.org/species/hg.html

References

1. Jurka J: **Repbase Update: a database and an electronic journal of repetitive elements**. *Trends Genet* 2000, **16**(9):418–420.
2. Altschul SF, Gish W, Miller W, Myers EW, Lipman DJ: **Basic local alignment search tool**. *J Mol Biol* 1990, **215**(3):403 – 410.
3. Al Ait L, Yamak Z, Morgenstern B: **DIALIGN at GOBICS—multiple sequence alignment using various sources of external information**. *Nucleic Acids Research* 2013, **41**(W1):W3–W7.
